# Draft genome sequence of a halophilic, putative hydrocarbon-degrading *Arhodomonas* sp. strain KWT2

**DOI:** 10.1128/mra.01023-24

**Published:** 2024-12-10

**Authors:** Damilare Ajagbe, William Marsh, Robert Murdoch, Babu Fathepure

**Affiliations:** 1 Department of Microbiology and Molecular Genetics, Oklahoma State University, Stillwater, Oklahoma, USA; 2 Biosciences Division, Battelle Memorial Institute, Columbus, Ohio, USA; University of Southern California, Los Angeles, California, USA

**Keywords:** halophiles, *Arhodomonas*, petroleum compounds, biodegradation, produced water

## Abstract

We report the draft genome sequence of a halophilic, putative hydrocarbon-degrading *Arhodomonas* sp. strain KWT2. The bacterium was isolated from a crude oil-contaminated saline pond in Kuwait. The genome is 3,923,868 bp in size and contains 3,534 predicted protein-coding genes, including genes for salt-tolerance and degradation of petroleum hydrocarbons.

## ANNOUNCEMENT

*Arhodomonas* sp. strain KWT2 was isolated from a crude oil-contaminated saline pond in Kuwait (29.1 N 48.0 E). Water samples were collected in sterile containers and stored at 4°C until use. The bacterium was enriched in 160 mL bottles containing 50 mL of the pond water, spiked with 2 µL of each BTEX (Benzene, Toluene, Ethylbenzene, and Xylene) compound as the carbon source. Bottles were closed with Teflon-coated septa and aluminum crimps. BTEX degradation was monitored using a gas chromatograph (1). The bottles were repeatedly spiked with BTEX, allowing for complete degradation before reintroducing BTEX. The culture was replenished monthly for a period of 4 months with 50% fresh mineral salts medium (MSM) containing 2.5 M NaCl until a stable enrichment that degraded BTEX within 7 days was obtained. To obtain a pure culture, the enrichment was plated on MSM agar plates containing 1 M NaCl. The plates were incubated in a desiccator with an open vial containing 1 mL of BTEX until colonies appeared (2). Distinctly isolated colonies were purified by replating twp times and identified using 16S rRNA-gene amplification (3). 16S rRNA-gene sequence of the isolate was compared to sequences in NCBI using BLASTn and found that strain KWT2 is 99.55% identical to *Arhodomonas recens* (NR_118045.1).

The genomic DNA was isolated using the DNeasy Ultraclean Microbial Kit (Qiagen, MD, USA), following the manufacturer’s instructions from cells grown in MSM containing 5 mM acetate and 1 M NaCl at 30 °C. The library preparation and sequencing were done using the PE 150 NovaSeq platform by Novogene (Sacramento, CA, USA). The sequencing generated 8,901,286 raw reads. For all analyses, default parameters were used for all softwares unless otherwise stated. Using Fastp v0.20.0 (4), the raw reads were processed for quality control by removing barcodes and low-quality reads. After filtering, 8,864,626 reads (99.59%) remained, and the quality of the clean reads was assessed using FastQC v0.1.12.1 (5).

Bioinformatic analyses were carried out using KBase (6). The reads were assembled using MEGAHIT v1.2.9 (7), with a minimum contig length of 1,000 bp, and the assembly quality was assessed using Quast v4.4 (8). The assembly yielded 53 contigs with *N*_50_ of 144,811 bp and L50 of 8 containing the largest contig length of 516,930 bp. The total length of the assembly was 3,923, 868 bp with G + C content of 69.47% and average depth of 338.8×. CheckM analysis v1.0.18 (9) indicated a genome completeness score of 99.12% and 0.88% contamination.

Gene calling and annotation of the genome were performed using the NCBI Prokaryotic Genome Annotation Pipeline v6.8 (10) resulting in 3,534 predicted protein-coding genes, 5 rRNAs, and 45 tRNAs. Functional characterization was done using Ghost KOALA v2.0 (11) and eggNOG mapper v2.1.12 (12) for KO and COG assignments, respectively. The encoded KEGG pathways were visualized using the KEGG Mapper-Reconstruct tool v5.2 (13). The genome of strain KWT2 will advance our understanding of the molecular mechanisms underlying hydrocarbon degradation in high-salinity environments.

## Data Availability

The genome sequence is deposited under the GenBank accession number JBHFPQ000000000. The BioProject and Biosample accession numbers are PRJNA1158845 and SAMN43552202, respectively. The Sequence Read Archive information is available under the accession number SRR30676589.
